# Nanoparticles Alter Secondary Metabolism in Plants via ROS Burst

**DOI:** 10.3389/fpls.2017.00832

**Published:** 2017-05-19

**Authors:** Gregory Marslin, Caroline J. Sheeba, Gregory Franklin

**Affiliations:** ^1^Chinese–German Joint Laboratory for Natural Product Research, Qinling-Bashan Mountains Bioresources Comprehensive Development C.I.C., College of Biological Science and Engineering, Shaanxi University of TechnologyHanzhong, China; ^2^Randall Division of Cell and Molecular Biophysics, King’s College LondonLondon, United Kingdom; ^3^Department of Integrative Plant Biology, Institute of Plant Genetics, Polish Academy of SciencesPoznan, Poland

**Keywords:** nanoparticles, nanopollution, reactive oxygen species, antioxidant enzymes, signaling pathways, plant secondary metabolism

## Abstract

The particles within the size range of 1 and 100 nm are known as nanoparticles (NPs). NP-containing wastes released from household, industrial and medical products are emerging as a new threat to the environment. Plants, being fixed to the two major environmental sinks where NPs accumulate — namely water and soil, cannot escape the impact of nanopollution. Recent studies have shown that plant growth, development and physiology are significantly affected by NPs. But, the effect of NPs on plant secondary metabolism is still obscure. The induction of reactive oxygen species (ROS) following interactions with NPs has been observed consistently across plant species. Taking into account the existing link between ROS and secondary signaling messengers that lead to transcriptional regulation of secondary metabolism, in this perspective we put forward the argument that ROS induced in plants upon their interaction with NPs will likely interfere with plant secondary metabolism. As plant secondary metabolites play vital roles in plant performance, communication, and adaptation, a comprehensive understanding of plant secondary metabolism in response to NPs is an utmost priority.

## Introduction

The National Science Foundation (NSF) projects that the global market for products incorporating nanotechnology could amount to three trillion USD by 2020 (Roco, 2011). Currently, more than 1000 commercial products containing nanoparticles (NPs) are available in the market (Vance et al., 2015). The NPs commonly found in household, industrial and healthcare products are Au (Gold), Ag (silver), ZnO (zinc oxide), CuO (copper oxide), TiO_2_ (titanium dioxide), Fe_3_O_4_/Fe_2_O_3_ (iron oxides), and CeO_2_ (cerium oxide). Similarly, incorporation of Ag, ZnO, TiO_2_, and SiO_2_ (silicon dioxide) NPs into agrochemicals (pesticides, fungicides, herbicides, fertilizers, etc.) is expected to have great potential in nanotechnology-driven smart agriculture (DeRosa et al., 2010; Khot et al., 2012; Parisi et al., 2015; Boxi et al., 2016; Fraceto et al., 2016). The expanding applications of nanotechnology in domestic, industrial and agricultural sectors are also increasing the possibilities of NPs reaching the environment as nanomaterial-containing wastes. As the consequences of NP pollutants reaching the environment in significant quantities are unknown, understanding the plant’s response to NPs is an intensive area of research.

Most studies with NPs indicated a certain degree of phytotoxicity, especially at high concentrations (Miralles et al., 2012). Depending on their size, NPs can enter plant cells from the apoplast, crossing the plasma membrane via endocytosis; subsequently they can be translocated from one part to another through symplastic flow (Rico et al., 2011). There is also evidence for the transport of NPs into subcellular organelles such as the nucleus, plastids, and vacuoles (Chichiriccó and Poma, 2015; Da Costa and Sharma, 2016).

*Arabidopsis thaliana* (L.) Heynh seedlings grown on soil treated with ZnONPs were observed to have reduced growth, chlorophyll content and rates of photosynthesis (Wang et al., 2016). These effects were concentration dependent with growth compromised 20 and 80%, respectively, with 200 and 300 mg/L treatments. At 300 mg/L, the chlorophyll content, net rate of photosynthesis, leaf stomatal conductance, intercellular CO_2_ concentration and transpiration rate were all reduced more than 50%. Similarly, an increasing concentration (0, 2.5, 10, 50, 100, and 1,000 mg/L) of CuONPs negatively affected *Oryza sativa* L. seedling growth in a hydroponic system (Da Costa and Sharma, 2016). Photosynthetic rate, transpiration rate, stomatal conductance, maximal quantum yield of PSII photochemistry, and photosynthetic pigment contents declined, with a complete loss of PSII photochemical quenching at 1,000 mg/L. ZnONPs inhibited the expression of genes involved in chlorophyll synthesis and photosystem structure (Wang et al., 2016). Accumulation of CuONPs in the chloroplasts was accompanied by a lower number of thylakoids per granum (Da Costa and Sharma, 2016). AgNPs inhibited Ribulose-1,5-bisphosphate carboxylase/oxygenase (Rubisco) activity and the photo-protective capacity of PSII in the model aquatic higher plant *Spirodela polyrhiza* (L.) Schleid (Jiang et al., 2017).

In addition to reduced photosynthetic rates the growth inhibition caused by NPs has also been associated with increased oxidative stress (Da Costa and Sharma, 2016; Li et al., 2016; Jiang et al., 2017). However, whether the arrest of photosynthesis or the induction of oxidative stress is the dominant impact of NPs is a subject of debate, since both of them go hand in hand (Aarti et al., 2006). Although the accumulation of NPs in chloroplasts and damage to the photosynthetic apparatus (Da Costa and Sharma, 2016; Jiang et al., 2017) supports the former, the fact that to reach the chloroplast NPs must cross the plasma membrane, where they can induce reactive oxygen species (ROS) via NADPH oxidases (Sosan et al., 2016) argues the reverse. ROS production, damage to the membrane structure and function, and fluctuation in antioxidant enzymatic activities are documented across plant species as common responses to NPs (Thwala et al., 2013; Vannini et al., 2013; Fu et al., 2014; Mirzajani et al., 2014; Hossain et al., 2015; Xia et al., 2015; Jiang et al., 2017; Tripathi et al., 2017). A few studies have also demonstrated that treatment of plants and photosynthetic microorganisms with NPs resulted in increased production of phenolics (Comotto et al., 2014; Ghorbanpour and Hadian, 2015; Večeřová et al., 2016), which might act as antioxidants to scavenge the ROS (Dixon and Paiva, 1995; Franklin et al., 2009).

The possibility of NP-induced disturbance in ROS homeostasis and associated signaling pathways as a major factor underlying the changes in plant secondary metabolism is explored in this perspective.

## “Oxidative Stress”- A Common Response of Plant to NPs Treatment

Oxidative burst has been consistently reported in plants exposed to toxic levels of NPs (Thwala et al., 2013; Hossain et al., 2015; Xia et al., 2015). Exposure to various NPs, for example Ag, ZnO, and Al_2_O_3_ (aluminum oxide), also induced reactive nitrogen species (^∗^NO, nitric oxide) and H_2_O_2_ in duckweed (Thwala et al., 2013), corn (Zhao et al., 2012) and tobacco bright yellow (BY2) cells (Poborilova et al., 2013). In tobacco BY2 cells, Al_2_O_3_NPs also induced the production of superoxide anion (O⋅_2_^-^), one of the highly reactive forms of ROS. Although it is debated whether ROS activation stems, actually, from intact particles or, rather, from ions released from NPs, recent studies supports the latter. In *S. polyrhiza*, internalized Ag, regardless of whether the exposure was Ag^+^ ions or AgNPs, had the same capacity to generate ROS supporting the hypothesis that intracellular AgNPs dissociate into highly toxic Ag^+^ ions (Jiang et al., 2017). Similarly, dissolution of ZnO, CuO, and CeO_2_ (cerium oxide) into their respective ions (Zn^2+^, Cu^2+^, or Ce^4+^) has been established in other studies (Ebbs et al., 2016; Bradfield et al., 2017).

The mechanisms through which NPs induce ROS production and trigger oxidative stress at the cellular level have also been investigated. AgNPs triggered Ca^2+^ and ROS signaling through the induction of Ca^2+^-permeable pores and direct oxidation of apoplastic L-ascorbic acid (Sosan et al., 2016). *A. thaliana root hair defective 2* (*rhd2*) mutant lacking NADPH oxidase RBOHC showed a significantly lower level of ROS generation in response to AgNPs compared with wild type plants (Sosan et al., 2016), indicating that the accumulation of ROS in cells is mediated by plasma membrane-bound NADPH oxidases (RBOH) enzymes that produce ROS at the apoplast (Mittler, 2017). On the other hand, chloroplastic ROS generation was observed in *S. polyrhiza*, based on the ability of AgNPs to inhibit Ribulose-1,5-bisphosphate carboxylase/oxygenase (Rubisco) activity and the photo-protective capacity of PSII (Jiang et al., 2017).

A common consequence of harmful levels of ROS is the damage to cellular macromolecules including membrane lipids that leads to cell death (Van Breusegem and Dat, 2006). Growth inhibition coupled with lipid peroxidation has been reported in *O. sativa* seedlings treated with 0.5, 1.0, and 1.5 mM CuONPs (Shaw and Hossain, 2013) and in 5 mg/L TiO_2_NPs treated *Nitzschia closterium* (Xia et al., 2015). NPs could also damage other macromolecules like DNA. AgNPs and AuNPs affected cell division in *Allium cepa* L. root tip cells (Kumari et al., 2009; Rajeshwari et al., 2016), the former causing chromatin bridge, chromosomal stickiness, disturbed metaphase, multiple chromosomal breaks, and cell disintegration (Kumari et al., 2009). DNA damage, mitochondrial dysfunction, and cell apoptosis were also observed in eggplant, as a consequence of oxidative stress induced by Co_3_O_4_ (Faisal et al., 2016).

In order to mitigate the effects of oxidative stress plants activate both enzymatic and non-enzymatic antioxidant defense machinery to scavenge excess ROS (Sewelam et al., 2016). Correspondingly, NP-mediated stress also activates plant’s antioxidant machinery/enzymes. Briefly, superoxide dismutase (SOD) that catalyzes detoxification of O⋅_2_^-^ into either ordinary molecular oxygen (O_2_) or H_2_O_2_ and ascorbate peroxidase (APX), which detoxifies peroxides such as H_2_O_2_ using ascorbic acid (Asc) as a substrate, were up-regulated in plants upon treatment with NPs (Fu et al., 2014). Whereas, dehydroascorbate reductase (DHAR) and monodehydroascorbate reductase (MDAR) enzymes that regulate the cellular Asc redox state were downregulated (Fu et al., 2014). Proteomic analysis of AgNPs treated *O. sativa* roots revealed an increased abundance of SOD, APX, and glutathione-*S*-transferase (GST) (Mirzajani et al., 2014). These NPs also stimulated the activities of SOD and APX significantly, while inhibiting glutathione reductase (GR) and DHAR in *Pisum sativum* L. seedlings (Tripathi et al., 2017). Catalase (CAT), another enzyme that protects the cells from oxidative damage, was significantly elevated upon treatment of wheat roots with 500 mg/kg CuONPs (Dimkpa et al., 2012). Maize plants germinated and grown on soil amended with 0, 400, and 800 mg/kg CeO_2_NPs showed a concentration dependent increase in the accumulation of H_2_O_2_ when tested after 10 days, but on day 20 did not show any difference (Zhao et al., 2012). A similar pattern in the increase of CAT and APX activities protected CeO_2_NP treated maize seedlings from lipid peroxidation (Zhao et al., 2012).

As disruption of ROS homeostasis impairs plant growth and development, whereas maintenance of ROS levels within appropriate parameters promotes plant health (Mittler, 2017), it is emerging that the induction of antioxidant machinery by NPs might promote plant growth as reported in a few studies (Sharma et al., 2012; Burman et al., 2013; Kumar et al., 2013) as long as a harmful level of ROS is not reached in the cells, whereas, once breached, this may lead to impaired organelle function, membrane damage, and eventually phytotoxicity.

## “NP-Induced ROS”- Can It Be An Inductive Signal for Plant Secondary Metabolism?

So far, a handful of studies have showed that NPs could affect microbial and plant secondary metabolism. For example, the concentration of phenolic compounds secreted to an extracellular medium was increased 127.5 and 22.1%, respectively, in *Arthrospira platensis* Gomont (cyanobacterium) and *Haematococcus pluvialis* Flotow (microalga) after treating with 100 mg/L TiO_2_NPs (Comotto et al., 2014). Artemisinin content was increased 3.9-fold in *Artemisia annua* L. hairy root cultures after 900 mg/L AgNPs treatment for 20 days (Zhang et al., 2013). This increase was associated with oxidative stress (H_2_O_2_ production), lipid peroxidation and CAT activity. A substantial increase in plant growth and diosgenin concentration was observed in fenugreek after 2 μg/kg AgNP treatment (Jasim et al., 2017). Ferulic acid and isovitexin were increased in barley plants exposed to CdO (cadmium oxide) NPs in air for 3 weeks at a concentration of 2.03 ± 0.45 × 10^5^ particles cm^-3^ (Večeřová et al., 2016). In *A. thaliana*, anthocyanin and flavonoid biosynthetic genes were upregulated in response to AgNPs (Garcia-Sanchez et al., 2015).

Although all the studies discussed above provide evidence for NP-mediated modulation of plant secondary metabolism, the following studies provide an indirect link between ROS and secondary metabolism. *Satureja khuzestanica* Jamzad calli growth improved significantly with increasing concentrations of carbon nanotubes (CNTs) in culture medium up to 50 mg/L, and then began to decrease at 500 mg/L (Ghorbanpour and Hadian, 2015). At this toxic concentration (500 mg/L), the highest level of H_2_O_2_ was observed together with significantly higher polyphenol oxidase (PPO), peroxidase (POD), and secondary metabolic activities. Similarly, when *A. thaliana* was exposed to 250 and 1000 mg/L CeO_2_ and indium oxide (In_2_O_3_) NPs, in addition to excessive ROS production, the activities of phenylalanine ammonia lyase (PAL) and PPO were greatly induced (Ma et al., 2016) revealing a possible role of secondary metabolism in protection against oxidative stress. Furthermore, PAL is the first enzyme of the general phenylpropanoid pathway that catalyses the deamination of phenylalanine to cinnamic acid and play a key role in diverting aromatic amino acids from primary metabolism to phenylpropanoid pathway.

There are several lines of evidence available in the literature implicating ROS-mediated signaling events as inductive cues for plant secondary metabolism. ROS themselves are signaling molecules, capable of inducing plant secondary metabolism (Simon et al., 2010). This could be observed during the wound-induced activation of secondary metabolism where ROS plays a key role as signaling molecule (Jacobo-Velazquez et al., 2015). In addition, ROS can also serve as signals for other messengers like jasmonic acid (JA) (Wu and Ge, 2004), salicylic acid (SA) (Maruta et al., 2012; Noshi et al., 2012; Wrzaczek et al., 2013; Baxter et al., 2014), ethylene (ET) (Zhang et al., 2016a,b), NO (Wang et al., 2013; Lindermayr and Durner, 2015), brassinosteroids (BRs) (Xia et al., 2009), etc., which are capable of modulating secondary metabolisms directly or indirectly.

To support the notion that ROS induced by NPs acts as signals for secondary metabolism, many indirect lines of evidence are available. ZnONP treatment induced SA, whereas it suppressed JA in *A. thaliana* (Vankova et al., 2017). Moreover, SA-mediated systemic acquired resistance (SAR) against microbial pathogens was compromised in *A. thaliana* after treatment with Ag, TiO_2_NPs, and CNTs, resulting in an increased colonization by *Pseudomonas syringae* pv. tomato, *Pst* (Garcia-Sanchez et al., 2015). These authors further suggested that SA pathway repression is a common feature of NP exposure, as an inducible kinase in the pathway that activates basal immune response upon perception of bacterial flagellin namely FLG22-induced receptor-like kinase 1 (FRK1) was downregulated in response to NPs (Garcia-Sanchez et al., 2015). In addition to SA-mediated SAR, other signaling pathways such as ET, BRs, and NO were also affected by NPs. In *A. thaliana* plants treated with AgNPs expression of ET biosynthetic components 1-aminocyclopropane-1-carboxylate synthase ACC and ACC oxidase 2 was reduced (Syu et al., 2014), suggesting that these NPs could inhibit ET perception and affect its biosynthesis. ET is an important signaling molecule mediating sesquiterpenoid biosynthesis in the *Atractylodes lancea* (Thunb.) endophytic fungi *Gilmaniella* sp. AL12 interaction (Yuan et al., 2016). BRs, the steroidal phytohormones that play important role in plant growth, secondary metabolite accumulation, stress responses and adaptation (Çoban and Göktürk Baydar, 2016) could ameliorate ZnONP-induced oxidative stress by improving antioxidant potential and redox homeostasis in tomato seedlings (Li et al., 2016). NO, another universal signaling molecule that plays a central role in secondary metabolite production in plant cells (Zhang et al., 2012; Zeng et al., 2014), is also involved in plant–NP interactions. For instance, AgNP-induced phytotoxicity could be alleviated by NO in *P. sativum* seedlings (Tripathi et al., 2017). Correspondingly, *O. sativa* NO excess mutant (noe1) plants were tolerant to ZnONP treatment, whereas OsNOA1-silenced (noa1) plants were susceptible to ZnONP-induced phytotoxicity (Chen et al., 2015).

## Possible Mechanisms of Modulation of Plant Secondary Metabolism By NPs

**FIGURE 1:**
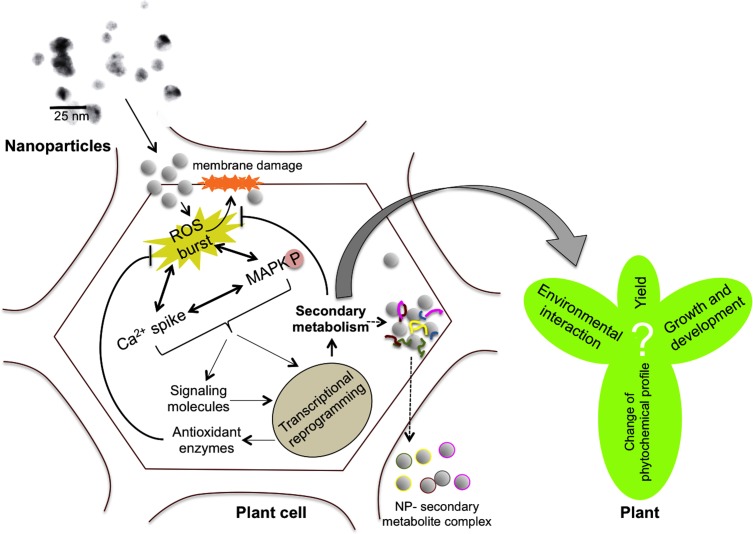
**Scheme describing the possible mechanisms involved in nanoparticle (NP)-mediated modulation of plant secondary metabolism.** NPs could enter plant cells through plasmodesmata causing physical damage to the plasma membrane. NPs can induce reactive oxygen species (ROS) production, calcium spikes, antioxidant machinery activation, mitogen-activated protein kinase (MAPK) cascades, etc., which could lead to transcriptional reprogramming of secondary metabolism. Activation of enzymatic antioxidant and non-enzymatic machineries including secondary metabolism might scavenge the ROS and protect the cells from oxidative damage. However, the exact consequences of changes in plant secondary metabolism on plant’s performance, environmental interaction, growth and yield are still unknown. On the other hand, the ability of NPs (e.g., anatase TiO_2_) to enter plant cells and exit as NP-secondary metabolite complexes could possibly be exploited for molecular pharming (dotted arrows).

Although the aforementioned reports suggest that NPs are interfering with various signaling pathways and capable of modulating plant secondary metabolism, the exact mechanism through which this modulation could occur is not understood. We believe that the initial responses of plants to NPs might include elevated levels of ROS, cytoplasmic Ca^2+^ and upregulation of mitogen-activated protein kinase (MAPK) cascades similar to other abiotic stresses (**Figure 1**) because of the following reasons. Recognition of AgNPs by plasma membrane bound receptors triggered a Ca^2+^ burst and ROS induction in *A. thaliana* (Sosan et al., 2016). Ca^2+^ levels and associated signaling pathway proteins were found to be upregulated in the proteomic analysis of AgNP treated *O. sativa* roots (Mirzajani et al., 2014). These authors hypothesized that AgNPs, or ions released thereof, impede cell metabolism by binding to Ca^2+^ receptors, Ca^2+^ channels, and Ca^2+^/Na^+^ ATPases. As sensed by calcium binding proteins (CaBPs) or other NP-specific proteins, NPs either mimic Ca^2+^ or signaling molecules in the cytosol (Khan et al., 2017). MAPK phosphorylation, and activation of downstream transcription factors generally lead to the transcriptional reprogramming of secondary metabolism in plants (Vasconsuelo and Boland, 2007; Schluttenhofer and Yuan, 2015; Phukan et al., 2016). Although no direct evidence for the involvement of MAPK pathways in plant-NP interactions is available, animal and human cell line studies revealed that analogous pathways are involved in AgNP-induced signaling (Eom and Choi, 2010; Lim et al., 2012), and it has been postulated that plants may also utilize MAPK cascade upon exposure to Ag NPs (Kohan-Baghkheirati and Geisler-Lee, 2015).

## Conclusion

As discussed in this article, exposure to NPs has the potential to alter plant secondary metabolism. Secondary metabolites can act as phytoalexins/phytoanticipins to protect plants from herbivores and pathogenic microbes, as signals for plant symbiotic interactions with beneficial microbes and as allelopathic agents to protect plants from rhizosphere competitors (Abdel-Lateif et al., 2012). In addition, they also serve as physical and chemical barriers to abiotic stressors and as antioxidants to scavenge ROS (Franklin et al., 2009; Ramakrishna and Ravishankar, 2011). Although NP-mediated changes in plant secondary metabolism would affect the optimal interaction of plants with their surrounding environment and possibly growth and productivity, substantial research is needed to understand the exact impact.

The presence of NPs in the environment might affect the pharmacological properties of medicinal plants, as many phytomedicines exert their beneficial effects through additive or synergistic actions of several compounds acting on single or multiple target sites associated with a physiological process (Briskin, 2000). While it is necessary to tackle these adverse effects, NP-mediated changes in secondary metabolism could also be beneficial if harnessed in such a way that NPs are used as elicitors in molecular pharming to enhance the production of desired secondary metabolites. For example, the content of important drugs like artemisinin (Zhang et al., 2013) and diosgenin (Jasim et al., 2017) were enhanced in plants treated with NPs. The ability of NPs to adsorb secondary metabolites (Kurepa et al., 2014) could be exploited for purification of precious compounds from plants via nanotrapping, if harnessed properly. Similarly, *in vitro* green synthesis of NPs using plant extracts can be further extended to develop high throughput tools to purify specific classes of compounds, as green synthesized NPs are often found as conjugates of secondary metabolites (Marslin et al., 2015).

Paucity of knowledge on the exact consequences of NP accumulation in the environment on plant metabolism is exacerbated by the fact that most of the studies have been conducted under controlled laboratory conditions and typically at much higher concentrations than what could be expected in the environment (Gottschalk et al., 2009; Baalousha et al., 2016). For instance, to induce statistically significant changes in the growth characteristics of *A. thaliana* plants, the minimum concentration of AgNPs was 300 mg/L under laboratory conditions (Sosan et al., 2016), a value much higher compared to the predicted environmental concentration of AgNPs in different environmental compartments: e.g., 1.3–4.4 mg/kg in sewage sludge (Gottschalk et al., 2009; Choi et al., 2017). Moreover, the ecologically relevant concentration of NPs largely depends on their environmental fate, plant species, characteristics of NPs, the medium through which it reaches the plant, etc. (Yin et al., 2012; Syu et al., 2014; Goswami et al., 2017), in addition to other, yet unknown, parameters. Although a recent study showed that ecologically relevant size and concentration of CdONPs could activate secondary metabolism in barley plants (Večeřová et al., 2016), it is difficult to generalize the impact of NPs on plant secondary metabolism in the environmental perspective. However, it is necessary to improve our understanding on the environmental fate of NPs and their hazards/risks, testing ecologically relevant conditions and concentrations in the context of plant secondary metabolism. Considering that plant secondary metabolism includes a vast array of compounds that are tightly controlled by signaling events and environmental cues, a case-by-case analysis might be necessary to have a deeper understanding.

## Author Contributions

GM collected information on NPs and secondary metabolism. CS prepared the possible mechanisms of plant secondary metabolism induction by NPs. GF conceived the idea of this perspective and collected all other information on ROS, singling pathways, and phytotoxicity in response to NPs. All the authors participated in writing and approved the manuscript for publication.

## Conflict of Interest Statement

The authors declare that the research was conducted in the absence of any commercial or financial relationships that could be construed as a potential conflict of interest.
